# Combined Untargeted and Targeted Metabolomics Approaches Reveal Urinary Changes of Amino Acids and Energy Metabolism in Canine Babesiosis With Different Levels of Kidney Function

**DOI:** 10.3389/fmicb.2021.715701

**Published:** 2021-09-17

**Authors:** Josipa Kuleš, Ivana Rubić, Blanka Beer Ljubić, Petra Bilić, Renata Barić Rafaj, Mirna Brkljačić, Richard Burchmore, David Eckersall, Vladimir Mrljak

**Affiliations:** ^1^Laboratory of Proteomics, Internal Diseases Clinic, Faculty of Veterinary Medicine, University of Zagreb, Zagreb, Croatia; ^2^Internal Diseases Clinic, Faculty of Veterinary Medicine, University of Zagreb, Zagreb, Croatia; ^3^Department of Chemistry and Biochemistry, Faculty of Veterinary Medicine, University of Zagreb, Zagreb, Croatia; ^4^Glasgow Polyomics, Wolfson Wohl Cancer Research Centre, University of Glasgow, Glasgow, United Kingdom; ^5^College of Medical, Veterinary, and Life Sciences, Institute of Biodiversity, Animal Health, and Comparative Medicine, University of Glasgow, Glasgow, United Kingdom

**Keywords:** metabolomics, kidney function, babesiosis, zoonosis, urine metabolomics, mass spectrometry

## Abstract

Canine babesiosis is a tick-borne disease with a worldwide distribution, caused by the haemoprotozoan parasites of the genus *Babesia*. One of the most prevalent complication is acute kidney injury, and an early diagnosis of altered kidney function remains a challenge for veterinary practice. The aim of this study was to assess the urine metabolic profile from dogs with babesiosis and different degree of kidney function using untargeted and targeted MS-based metabolomics approaches. In this study, 22 dogs naturally infected with *Babesia canis* and 12 healthy dogs were included. Untargeted metabolomics approach identified 601 features with a differential abundance between the healthy group and groups of dogs with babesiosis and different level of kidney function, with 27 of them identified as a match to known standards; while targeted approach identified 17 metabolites with significantly different concentrations between the groups. A pattern of significantly altered metabolites referring to the inflammatory host response, oxidative stress, and energy metabolism modulation in babesiosis was presented. Our findings have demonstrated that kidney dysfunction accompanying canine babesiosis was associated with changes in amino acid metabolism, energy metabolism, fatty acid metabolism, and biochemical pathways such as urea cycle and ammonia detoxication. These findings will enable the inclusion of urinary markers for the detection and monitoring of renal damage in babesiosis, as well as in other similar diseases.

## Introduction

Babesiosis is one of the most important vector-borne zoonoses worldwide. It is a widespread haemoprotozoan disease with a considerable global economic and health impact that can infect various vertebrate hosts, including humans (Bilić et al., 2018). The main cause of canine babesiosis in Europe is *Babesia canis*. Canine *B. canis* infection has been reported in different European countries and sporadically around the world, with the prevalence ranging from 2.3 up to 44.8% (Bilić et al., 2018). Global presence and quick spreading of babesiosis is on the account of the expansion of tick habitats and increased mobility of animals.

Acute kidney injury (AKI), recognized as one of the most prevalent complication of canine babesiosis, causes azotemia and uremia in dogs due to a decrease of the glomerular filtration rate (Lobetti et al., 1996; Máthé et al., 2007). Anemia and systemic hypotension have been identified as the primary causes of renal hypoxia leading to the renal damage in babesiosis (Jacobson et al., 2000; Zygner and Gójska-Zygner, 2014). Early diagnosis and monitoring of an altered kidney function remains a challenge in clinical veterinary practice, and there is a need for the identification of novel markers for an early and site-specific detection of kidney dysfunction.

There are different guidelines for the classification and grading of severity of renal damage in dogs. The International Renal Interest Society (IRIS) staging system for AKI was developed as a consensus scheme to promote a more uniform characterization and recognition of AKI in animals, while the Consensus Statements of the American College of Veterinary Internal Medicine (ACVIM) also provide guidelines for assessing and managing dogs and cats with renal proteinuria (Lees et al., 2005). According to these guidelines, significant proteinuria for dogs is manifested with a urine protein to creatinine ratio (UPC) > 0.5, with borderline proteinuria if the UPC is 0.2–0.5. In azotemic dogs, a UPC > 1 is associated with an increased risk of uremic crisis and death. Prospective monitoring is recommended for non-azotemic dogs with persistent microalbuminuria and non-azotemic dogs with persistent renal proteinuria and UPC values > 0.5, while diagnostic investigation and therapeutic intervention is required for non-azotemic dogs with persistent renal proteinuria and UPC values > 1.0, and dogs with azotemia and UPC values > 0.5 (Lees et al., 2005).

Metabolomics is the study of the metabolic profiles in a given biological setting, and it is one of the building blocks of systems biology that starts with activated genes (genomics) along with gene transcripts (transcriptomics) and proteins (proteomics) (Dettmer et al., 2007). Metabolites are small molecules that are chemically transformed during metabolism and, therefore, serve as direct signatures of biochemical activity. As they provide a functional readout of cellular state, changes at the metabolite level often appear in biofluids before the onset of clinical symptoms and they are, therefore, easier to correlate with the phenotype (Patti et al., 2012; Khamis et al., 2017).

There are two main analytical approaches in performing metabolomics studies: untargeted and targeted (Dettmer et al., 2007; Patti et al., 2012). The untargeted approach (metabolite fingerprinting, chemometric approach) compares the metabolic profiles between samples in order to detect changes in response to some internal or external factors (such as disease, diet, and drug development) providing only their relative quantification. The major challenge in untargeted metabolomics is metabolite identification, due to technological limitations such as analytical coverage of the platform used, the possible bias toward the detection of the most abundant molecules, as well as different fragmentation pattern of the same metabolite depending on the specific instrument or technique used. The targeted approach (metabolic profiling, quantitative approach) refers to the identification and quantification of a preselected category of metabolites within a sample. A limitation of this approach is the requirement for available purified standards.

Due to the chemical and physical diversity of metabolites, it is difficult to acquire a highly informative profiling of the whole metabolome. Therefore, for a more comprehensive and accurate metabolic profile, integrative implementation of two or more approaches are needed. For these reasons, our study combines the untargeted and targeted MS-based metabolomics approaches.

Urine is a convenient and key biological matrix for metabolome biomarker discovery. Advantages of using urine as a sample for metabolomics studies include the following: non-invasive and simple collection, metabolites richness, and ability to reflect metabolic dysregulation (Want et al., 2010). The analytical challenges regarding urine as a sample include its large variations in ionic strength, pH, osmolarity, and urine volume, as well as a huge dynamic range of metabolite concentrations, and an extreme diversity of chemical classes in urine. Variation in urine volume represents an analytical challenge requiring normalization, with the most common normalization strategies using creatinine concentration, specific gravity, and osmolality (Ryan et al., 2011; Khamis et al., 2017). In the absence of kidney diseases, creatinine is excreted in measurable and relatively constant amounts representing the glomerular filtration and active tubular excretion of the kidney. In contrast to creatinine, urine osmolality is not affected by diurnal rhythms, diet, activity, age, gender, stress, or health, as osmolite concentration is a reflection of the total endogenous metabolic turnout (Warrack et al., 2009).

Veterinary research is gaining an increasing interest in the metabolomics area, but it is still underexplored compared to the human medicine field (Carlos et al., 2020). Metabolomics strategies have a wide canine application area, as evidenced by previous studies investigating different diseases, such as heart disease, diabetes, hypothyroidism, chronic kidney disease (CKD), and others (Li et al., 2015; Forster et al., 2018; Ferlizza et al., 2020; Muñoz-Prieto et al., 2021). This is the first study to use metabolomics approaches to identify the urinary metabolomics profile in canine babesiosis. We hypothesized that renal involvement would progressively affect the urinary excretion of metabolites. The aim of this study was to assess the urine metabolic profile from dogs with babesiosis and different degree of kidney function using untargeted and targeted MS-based metabolomics approaches. Additionally, identifying the unique metabolic signatures and differences through different onset of disease is a useful tool to better understand the pathophysiology of babesiosis.

## Materials and Methods

### Study Design

In this study, 22 dogs naturally infected with *B. canis* and 12 healthy dogs were included. Dogs with babesiosis were divided into three groups: group A consisted of six non-azotemic dogs (serum creatinine < 140 μmol/L) with a normal urine protein to creatinine ratio (UPC < 0.5); group B of 10 non-azotemic dogs with UPC > 0.5; and group C of six azotemic dogs (serum creatinine > 140 μmol/L) with UPC > 1. Urine samples were analysed by untargeted hydrophilic interaction chromatography-mass spectrometry (HILIC-MS) and targeted liquid chromatography (LC) and flow injection analysis (FIA) coupled to tandem mass spectrometry (MS/MS) to obtain the urinary metabolomic profiles and pathways associated with canine babesiosis accompanied with kidney (dys)function.

### Animals

This study was approved by the Committee on Ethics of the University of Zagreb, Faculty of Veterinary Medicine (Permit Number: 640-01/18-17/63). Blood and urine samples were collected prior to treatment from dogs admitted to the Clinic for Internal Diseases, Faculty of Veterinary Medicine, University of Zagreb, Croatia. The diagnosis of babesiosis was made by the demonstration of parasites within the infected erythrocytes in thin blood smears stained with May-Grünwald-Giemsa stain (Merck, Darmstadt, Germany), while confirmation and subspecies determination were performed using PCR, as described previously (Beck et al., 2009). All the dogs have been subcutaneously administered with one dose (6 mg/kg) of imidocarb dipropionate (Imizol^®^12%, Schering-Plough, Kenilworth, NJ, United States) on the day of admission.

There were 22 dogs of different breeds with naturally occurring babesiosis caused by *B. canis*, with 11 females and 11 males, and with a median age of 4 years (min–max: 1–14 years).

The control group consisted of 12 healthy dogs, six females and six males, with a median age of 3.5 years (1–10 years). For inclusion in the study, none of the healthy dogs had histories of recent illness. Routine hematologic and biochemical analyses with urinalysis were performed to ensure the health status of the animals. PCR was performed as for infected dogs, to rule out a subclinical infection.

### Experimental Procedures

#### Sample Preparation

Voided midstream urine samples from healthy dogs and dogs with babesiosis were collected into sterile tubes. After urinalysis, the remaining urine was centrifuged at 500 *g* for 10 min at 4°C, and the supernatant was aliquoted and stored at −80°C until analysis. Routine urinalysis including semiquantitative dipstick test (Multistix 10SG, Siemens) was performed on an automated analyser (CLINITEK Status + Analyzer, Bayer) and the microscopic sediment analysis at a high (400x) power field. Urine protein and creatinine concentrations were determined using commercial kits on an automated chemistry analyser (Architect c4000, Abbot, IL, United States).

Serum samples were also collected for biochemistry analyses performed on the biochemistry analyser (Architect c4000, Abbot, IL, United States), while C-reactive protein (CRP) was assessed by canine specific assay (Gentian Diagnostics ASA, Moss, Norway) on the same analyser. Hematological data was generated from the plasma samples using an automatic hematology analyser, Horiba ABX (Diagnostics, Montpellier, France).

#### Untargeted LC-MS/MS Analysis

##### Metabolite extraction

For the untargeted analysis, urine samples were normalized according to the osmolality prior the extraction. For metabolite extraction, 25 μl of each urine sample were subjected to chloroform/methanol/water (1:3:1, v/v/v) extraction (chloroform and methanol from Honeywell, Charlotte, SAD; LC/MS grade water: Merck, Darmstadt, Germany). Pooled sample was prepared by mixing 10 μl of each sample (control and disease), and 25 μl of pooled sample was also subjected to extraction. Matrix blank sample contained only the extraction solvent. All samples (urine samples, pooled samples, matrix blank) were subsequently vortexed and centrifuged (13,000 *g* for 5 min at 4°C). The supernatant was stored at −80°C until LC-MS/MS analysis.

##### LC-MS/MS analysis

The analysis was performed on a Dionex UltiMate 3000 RSLC system (Thermo Fisher Scientific, Germering, Germany) coupled to a Thermo Orbitrap Q Exactive (Thermo Fisher Scientific, Bremen, Germany). The metabolites were separated using hydrophilic interaction liquid chromatography (HILIC) with a ZIC-pHILIC column (150 mm × 4.6 mm, 5 μm column, Merck Sequant, Darmstadt, Germany). The column was maintained at 25°C, and samples were eluted with a linear gradient (A: 20 mM ammonium carbonate in water; B: 100% acetonitrile) from 80% A and 20% B to 95% A and 5% B. The flow rate was 0.3 ml/min. Orbitrap Q Exactive instrument was operated in positive and negative mode at mass resolution of 70,000 and the full scan of m/z range 70–1,050. The source voltage was +3.8 kV for the positive mode and −3.8 kV for the negative mode, sheath gas 40 (arbitrary units), auxiliary gas 5 (arbitrary units), and capillary temperature of 320°C. A standard mix (kindly provided by Glasgow Polyomics, United Kingdom) consisted of about 150 compounds (reference compounds for metabolite identification) and was run together with the samples. The quality control samples were extracts obtained from beer and human urine (kindly provided by Glasgow Polyomics, United Kingdom) that were used to check the signal reproducibility and the quality of the chromatography.

##### Data processing

Metabolomics data were processed using the Polyomics integrated Metabolomics Pipeline (PiMP) available at http://polyomics.mvls.gla.ac.uk (Gloaguen et al., 2017). Metabolites were identified based on the retention times/masses or masses of detected peaks matched to the standards, while others were annotated using the metabolite databases HMDB (The Human Metabolome Database) and/or KEGG (Kyoto Encyclopedia of Genes and Genomes) integrated within the PiMP software.

#### Targeted Metabolomics Analyses

##### Metabolite extraction

Urine concentrations of the metabolites were determined with the AbsoluteIDQ^®^ p400 HR Kit (Biocrates Life Sciences AG, Innsbruck, Austria), allowing the targeted analyses of up to 408 metabolites from 11 metabolite classes. Metabolite concentrations of each sample were determined in a single analysis, but two separate MS analytical runs, a combination of liquid chromatography and flow-injection analysis (FIA) coupled to tandem mass spectrometry (MS/MS). LC-MS analysis was used for the quantitation of amino acids and biogenic amines while FIA-MS was used to quantify all other metabolites such as acylcarnitines, cholesterol esters, glycerophospholipids, glycerides, sphingolipids, and hexoses.

The sample extraction was performed on the specific 96-well plate system for the protein-removal, internal standard normalization, and derivatization according to the instructions of the manufacturer. Briefly, 10 μl of urine samples, calibration standards, zero standards, and quality control samples were pipetted to the 96-well kit plate containing the internal standard mix. Samples were dried for 30 min using a vacuum manifold (Thermo Scientific, Waltham, SAD) and then derivatized with 50 μl of 5% derivatization solution of phenylisothiocyanate (PITC) (Sigma-Aldrich, St. Louis, SAD) in water:ethanol:pyridine at a ratio of 1:1:1 (ethanol: Honeywell, Charlotte, United States; pyridine: BDH PROLABO, United Kingdom). The plate was incubated for 20 min at room temperature and dried again for 60 min. Metabolites were extracted by the addition of 300 μl of 5 mM ammonium acetate (Sigma-Aldrich, St. Louis, SAD) solution in methanol and shaken at 450 rpm for 30 min at room temperature. The extracts were collected using a vacuum manifold for 2 min into a capture plate for the FIA-MS analysis. For the LC-MS analysis, a total of 150 μl from the capture plate was transferred and diluted with 150 μl of LC-MS grade water to another plate, while 250 μl of FIA mobile phase (10 ml ampule Biocrates FIA mobile phase in 290 ml of methanol) was added directly to each well of the original capture plate.

##### LC-MS/MS analysis

Metabolites extracts were analysed using a Dionex Ultimate 3000 RSLC system coupled to a Thermo Orbitrap Q Exactive mass spectrometer using electrospray ionization source. The samples were loaded on a Thermo p400 HR UHPLC column provided with the kit and the column temperature was maintained at 50°C. Metabolites were eluted using mobile phase A [0.2% formic acid (Sigma-Aldrich, St. Louis, SAD) in water] and mobile phase B [0.2% formic acid in acetonitrile (Honeywell, Charlotte, SAD)]. The injection volume was 5 μl in every run. The total run time was 5.81 min using a gradient of 0–95% of mobile phase B over 4 min and the flow rate was 0.8 ml/min. In the FIA-MS analysis, metabolites were eluted using the FIA mobile phase at 0.05 ml/min for the first 1.6 min, then the flow rate increased to 0.2 ml/min for 1.2 min, and then decreased back to 0.05 ml/min for the rest of the program. The instrument analysis was performed in the full scan acquisition mode on both the positive and negative modes for LC-MS and FIA-MS, and all parameters were set according to the guidelines from the Biocrates instructions.

##### Data processing

The quantification of the LC-MS metabolites was performed via the XCalibur Quan 4.1 software (Thermo Fisher Scientific, Waltham, SAD), based on a 7-point calibration curve and isotope labeled internal standards for most analyses and by confirming the correct peak retention times and peak area integration. On the other hand, FIA-MS analysis used a single point calibrator with the representative internal standards. Biocrates’ in-house MetIDQ software (Biocrates Life Sciences AG, Innsbruck, Austria) was applied for the data processing, quality assessment, and data export. Quality control samples were within the pre-defined tolerances of the method. In terms of quantification, “semi-quantitative” referred to the compounds whose specific standards were not commercially available and a verification of the accuracy was not possible by the manufacturer. “Quantitative with restrictions” referred to the compounds that had expected coefficient of determination (*R*^2^) < 0.99 for the calibration curves.

#### Statistical Analysis

Differences between the healthy and diseased dogs for biochemistry and urinalysis were assessed by Kruskal-Wallis test using the statistical software, GraphPad Prism 5 (GraphPad Software Inc., San Diego, CA, United States). Statistical analysis of the metabolomics data was performed using an open source metabolomics data processing tool MetaboAnalyst 4.0 by means of univariate and multivariate statistical approaches (Chong et al., 2019).

For untargeted metabolomics, all statistical analyses were performed on the combined positive and negative ion data sets, exported as a peak intensity table from PiMP. Missing values (1.6%) were replaced by 1/5 of the minimum positive value for each variable. The intensities of the extracted metabolites were log transformed and then mean centered and divided by the square root of the standard deviation of each variable (Pareto-scaling), to correct for heteroscedasticity, reduce the skewness of the data, and to reduce mask effects.

For targeted metabolomics, missing values (8.4%) were replaced by the estimated missing value using KNN (feature-wise). The concentrations of the metabolites were normalized by reference feature (osmolality), log-transformed, and Pareto scaled.

The overall differences in the metabolic profiles of the four groups (Control, Disease A, Disease B, and Disease C) were analysed by one-way analysis of variance (ANOVA) and Fisher’s least significant difference (Fisher’s LSD) *post hoc* test, partial least square-discriminant analysis (PLS-DA), variable importance on projection (VIP), hierarchical clustering analysis (HCA), and random forest analysis. Correlations between the metabolites identified by the targeted approach and UPC (conventional parameter) were calculated by Pearson’s correlation coefficient. Statistical significance was set at *p* < 0.05. On the basis of the quantitative values of the targeted metabolomics approach, receiver operating characteristic (ROC) curves were applied to investigate the clinical diagnostic potentials of these metabolites.

Pathway and enrichment analyses were performed using the joint significant metabolites identified by the untargeted and targeted approach. For the pathway analysis, hypergeometric test was used for the enrichment method, relative–betweeness centrality for topology analysis, and *Homo sapiens* for pathway library. Metabolite Set Enrichment Analysis (MSEA) of the significant metabolites were examined with the pathway-associated metabolite sets (SMPDB; 99 metabolite sets based on normal human metabolic pathways set as metabolite set library).

## Results

### Assessment of Biochemistry and Urinalysis

In this study, 12 healthy dogs and 22 dogs naturally infected with *B. canis* were included and divided into three groups based on their kidney function assessments. Control and case groups were age and sex matched. Selected biochemistry and urinalysis results are presented in Table 1. Significant differences between groups assessed by Kruskal-Wallis test (*p* < 0.05) were found for serum albumin, creatinine, bilirubin, urea, aspartate aminotransferase (AST), CRP, and UPC in urine, showing a consistency with the laboratory findings in babesiosis and different degree of kidney dysfunction.

**TABLE 1:** Serum and urinary parameters in non-azotemic dogs with babesiosis and normal urine protein to creatinine ratio (UPC < 0.5) (group A), non-azotemic dogs with babesiosis and UPC > 0.5 (group B), azotemic dogs with babesiosis and UPC > 1 (group C), and healthy dogs (control group).

Parameter (unit)	Group A (*N* = 6)	Group B (*N* = 10)	Group C (*N* = 6)	Control group (*N* = 12)	*P*-value
Total protein (g/L)	56.5 (53–62.3)	60.5 (56.8–62.3)	61 (48.5–69)	63 (48.5–69)	0.159
Albumin (g/L)	26 (24.8–29.8)	28.5 (26.5–29.8)	26 (22.3–27.3)	30.5 (29.2–33.8)	**0.003**
Creatinine (μmol/L)	84.5 (66.8–154.5)	82 (59.8–102.8)	170 (138.8–296)	70 (64.5–85)	**0.005**
Bilirubin (μmol/L)	7.1 (3.5–10.6)	8.7 (6.2–11.3)	20.4 (9.8–243.7)	2 (1.7–2.6)	**<0.001**
Urea (mmol/L)	4.85 (3.53–10.85)	6.1 (5.5–9.2)	19.6 (9.4–30.4)	5.3 (4.73–6.03)	**0.004**
ALT (U/L)	37 (30–57.5)	62 (45–80)	55.5 (39.8–64.5)	39 (36–48.8)	0.254
AST (U/L)	118 (33–141)	103.5 (65.5–168.5)	333 (140–396)	24 (19–48.5)	**0.014**
CRP (mg/L)	162.9 (91–191.5)	132.9 (105.4–191.5)	159.8 (91.6–186)	3.4 (2–4.2)	**<0.001**
Creatinine (urine) (mmol/L)	15.9 (9.4–22.7)	19.6 (8.1–29.4)	9.1 (4.5–14.7)	19.5 (13.4–30)	0.186
UPC	0.28 (0.20–0.44)	1.54 (1.29–4.56)	4.17 (2.98–4.83)	0.09 (0.06–0.13)	**<0.001**

*Data are presented as median (Q1–Q3). *P*-value for the Kruskal-Wallis test is given. ALT, alanine transaminase; AST, aspartate aminotransferase; CRP, C-reactive protein. Significant *p*-values (<0.05) are in bold.*

### Untargeted Metabolomics

Following processing using PiMP, features were annotated or identified on the basis of the mass and mass/retention time matched to known standards, respectively. A total of 58 unique compounds matched to a known standard. Uploaded data file to MetaboAnalyst contained 34 samples in four groups by 3,207 peaks (mz/rt) (Supplementary Table 1A). Both univariate and multivariate statistics were employed.

**FIGURE 1:**
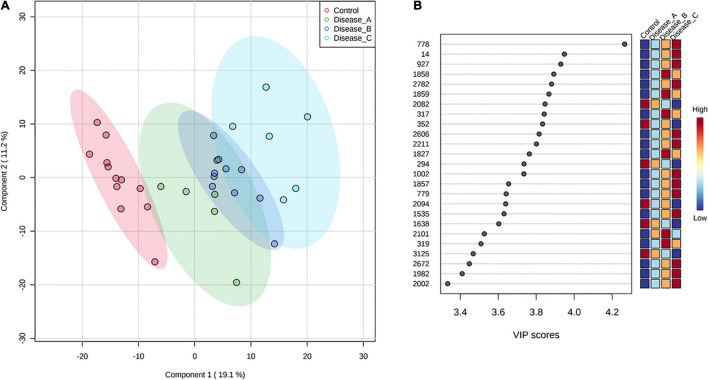
**(A)** Partial least squares discriminant analysis (PLS-DA) score plot of urine samples from the control group (red circle) and three groups of dogs with babesiosis and different degree of kidney function (A, green circle; B, dark blue; C, light blue) in the untargeted LC-MS based metabolomics approach. **(B)** Variable Importance in Projection (VIP) scores for the 25 most influential features of PLS-DA. Colored boxes on the right indicate the relative intensity of the corresponding feature in each group.

Partial least square-discriminant analysis allowed visualization of the data based on disease classification (Figure 1A). It showed a clear separation of dogs in the control group from dogs with babesiosis, but also a clear separation of group C from other groups, while groups A and B were mainly overlapping. The best classifier model comprised five components (*R*_2_ = 0.99, *Q*_2_ = 0.85), but the model with only one component also had a reasonable predictive value (*R*_2_ = 0.87, *Q*_2_ = 0.78).

Variable importance on projection scores for the 25 most influential features of PLS-DA showed a clear stepwise, mostly increase in the relative abundance of the corresponding features from the controls to the most affected group corresponding with the stages of kidney function (Figure 1B).

**FIGURE 2:**
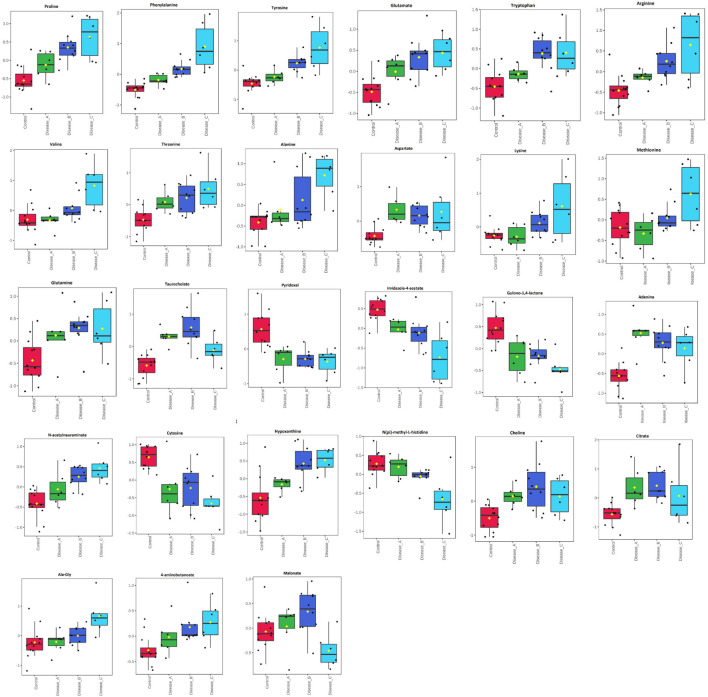
Comparison of the urinary metabolites identified by the untargeted metabolomics approach in non-azotemic dogs with babesiosis and normal urine protein to creatinine ratio (UPC < 0.5) (group A), non-azotemic dogs with babesiosis and UPC > 0.5 (group B), azotemic dogs with babesiosis and UPC > 1 (group C), and healthy dogs (control group). All metabolites shown demonstrated a statistically significant difference between groups by ANOVA (*p* < 0.05). Data are presented as box and whiskers plot (mean ± SD).

One-way analysis of variance and Fisher’s LSD *post hoc* test identified 601 features (identified and annotated) with a differential abundance between groups (Supplementary Table 1B). Among those, 27 were identified to the known standards, including amino acids (phenylalanine, proline, tyrosine, glutamate, tryptophan, and arginine, etc.), pyridoxal, adenine, *N*-acetylneuraminate, choline, citrate, and others (Figure 2 and Supplementary Table 1C). Increased urinary excretion in dogs with babesiosis was found for 13 amino acids. Urinary levels of two TCA cycle-associated metabolites, citrate and malonate, were significantly altered in our study. An interesting pattern was found for malonate levels, which were higher in group B compared to the control; however, group C showed significantly lower levels compared to groups A and B. Higher levels of purines, adenine, and hypoxanthine were noted in dogs with babesiosis, while a lower level of pyrimidine cytosine in dogs with babesiosis was observed compared to the control group. Taurocholate excretion was higher in all diseased groups, but of note is that excretion was higher in group B compared to group C. Urinary pyridoxal was lower in all groups with babesiosis compared to the control group, as well as L-gulono-1,4-lactone (GUL). Higher levels of choline were found in all groups of dogs with babesiosis compared to the controls. *N*-acetylneuraminic acid was higher in all groups of dogs with babesiosis compared to the control group, as well as in group C when compared to group A.

**FIGURE 3:**
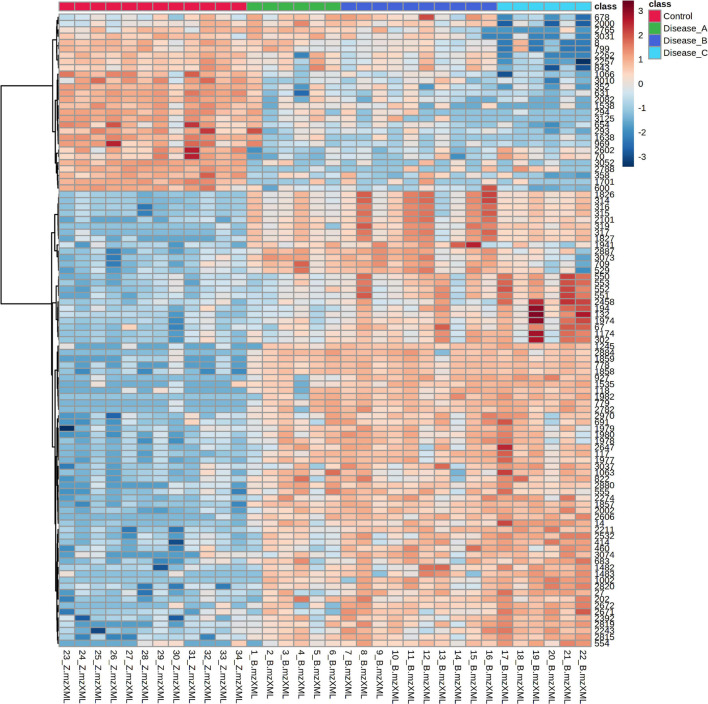
Hierarchical cluster analysis (HCA) based on the top 100 significant different features obtained with the untargeted metabolomics approach using ANOVA, among the control group (red panel) and three groups of dogs with babesiosis and different degree of kidney function (A, green panel; B, dark blue; C, light blue). Each colored cell on the map corresponds to the intensity value, with the red color meaning increased, and blue, a decreased level. Data was log transformed and Pareto-scaled.

The distribution of the most significant features separating the four groups was visualized by hierarchical cluster analysis using Euclidean as the distance measure, ward as clustering algorithm, and ANOVA for features ranking. Heat map showed a clear distinction of the control and affected groups, with more subtle changes between the affected groups (Figure 3).

**FIGURE 4:**
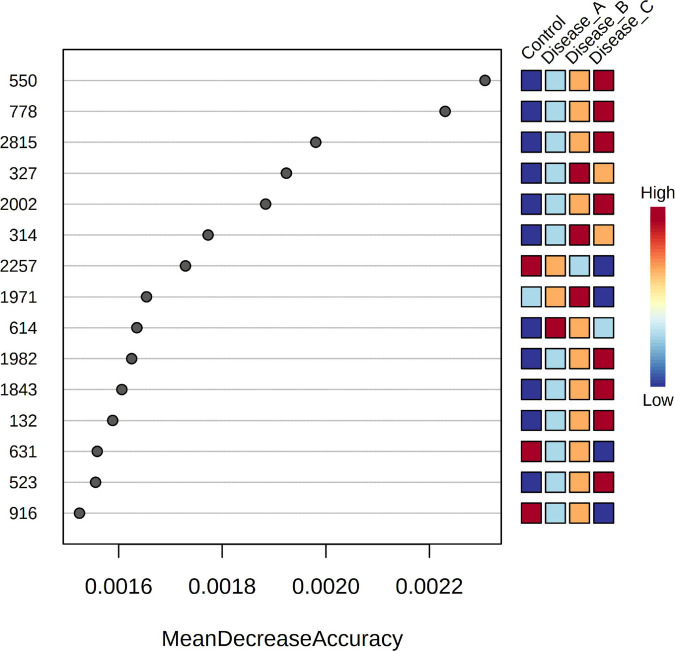
Random Forest variable importance plot for the significant features obtained by the untargeted metabolomics approach. A higher value indicates the importance of that metabolite in predicting groups.

Random forest (RF) analysis identified the features that had the highest discriminatory power between the four groups, using mean decrease accuracy as the measure of the performance of the model without each metabolite. A higher value indicates the importance of that feature in predicting groups (Figure 4). Random forest classification showed an excellent prediction of the control group, while classification of the affected groups showed less accuracy with a 35.3% out of bag (OOB) error rate. One of the discriminatory features, feature 132, was identified as phenylalanine. VIP scores of PLS-DA and RF ranked the features in terms of their ability to facilitate clustering and discrimination between groups, and in untargeted metabolomics, overlap was found for features 778, 2002, and 1982.

### Targeted Metabolomics

For targeted metabolomics analyses using the Biocrates kit, metabolites were quantified according to the protocol of the manufacturer using the MetIDQ^TM^ software for targeted metabolomics data processing and management.

The raw data were cleaned applying a 2/3 rule to exclude the metabolites below the limit of detection (LOD): at least 2/3 of the valid values above LOD needed to be available per analyte in the samples for each group for the statistical analysis. This reduced the dataset to 42 analytes. In total, 21 were fully validated as absolutely quantitative, 20 as semi-quantitative, and 1 quantitative with restrictions (Supplementary Table 2A). As the kit is primarily intended for use with human plasma samples, some analytes were out of reference range for urine (e.g., creatinine). All valid data was uploaded to MetaboAnalyst for further statistical analysis.

**FIGURE 5:**
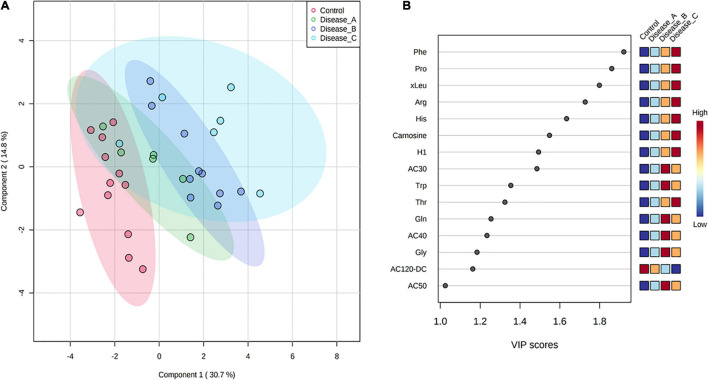
**(A)** Partial least squares discriminant analysis score plot of urine samples from the control group (red circle) and three groups of dogs with babesiosis and different degree of kidney function (A, green circle; B, dark blue; C, light blue) in the targeted MS-based metabolomics approach. **(B)** Variable Importance in Projection scores for the 25 most influential metabolites of PLS-DA. Colored boxes on the right indicate the relative concentration of the corresponding metabolite in each group.

Supervised PLS-DA indicated that the best classifier model comprised three components (*R*_2_ = 0.85, *Q*_2_ = 0.75; Figure 5A). This analysis showed a relatively clear separation of the group with healthy dogs from dogs with babesiosis, but, at the same time, showing the overlap of the three groups with disease.

Variable importance on projection scores for the 15 most influential features of PLS-DA demonstrated differences in the concentration of the corresponding metabolites from the controls to the affected groups, mostly showing a gradual increase of concentration corresponding with the stages of kidney dysfunction, and revealing amino acids as the five most influential metabolites [phenylalanine, proline, (iso)leucine, arginine, and histidine] (Figure 5B).

**FIGURE 6:**
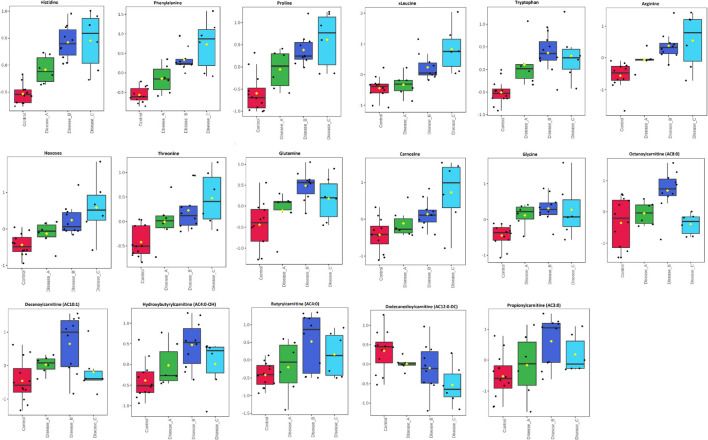
Comparison of the urinary metabolites identified by the targeted metabolomics approach in non-azotemic dogs with babesiosis and normal urine protein to creatinine ratio (UPC < 0.5) (group A), non-azotemic dogs with babesiosis and UPC > 0.5 (group B), azotemic dogs with babesiosis and UPC > 1 (group C), and healthy dogs (control group). All metabolites shown demonstrated a statistically significant difference between groups by ANOVA (*p* < 0.05). Data are presented as box and whiskers plot (mean ± SD).

One-way ANOVA and Fisher’s LSD *post hoc* test identified 17 metabolites with significantly different concentrations between groups, including different amino acids (e.g., histidine, phenylalanine, proline, and glutamine), hexoses (H1), carnosine, and acylcarnitines (Figure 6 and Supplementary Table 2B). Urinary excretion of nine amino acids were altered in babesiosis, with six of them identified by both approaches, untargeted and targeted (Phe, Pro, Trp, Arg, Thr, and Gln). Increased excretion of low-chain acylcarnitines (AC) (AC3:0, AC4:0, AC4:0-0H) and medium-chain AC (AC8:0, AC10:1) and decreased excretion of medium-chain, AC12:0-DC, were found in dogs with babesiosis. It is of interest that there were no differences between group A and the control group and that the most profound differences were found between group B and the control group for all increased ACs.

**FIGURE 7:**
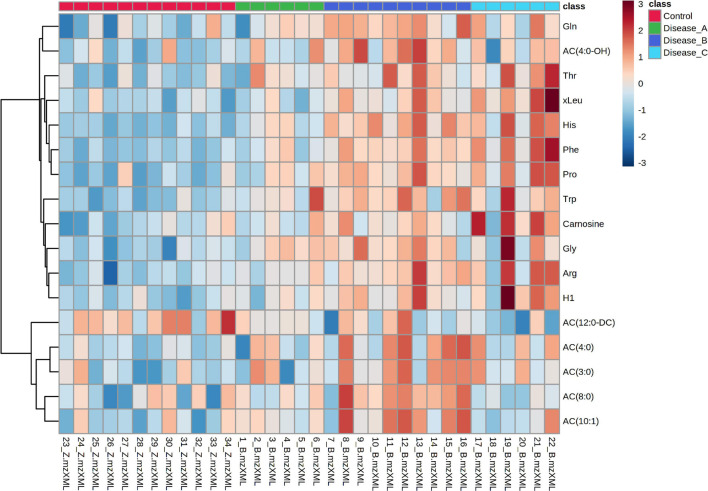
Hierarchical cluster analysis based on the significant different features obtained with the targeted metabolomics approach using ANOVA among the control group (red panel) and three groups of dogs with babesiosis and different degree of kidney function (A, green panel; B, dark blue; C, light blue). Data were normalized to osmolality, log transformed, and Pareto-scaled.

HCA was performed using the significant metabolites from ANOVA to obtain a visualization of the different phenotypes among the groups. A clear distinction of the control and affected groups was shown, while groups B and C showed a similar pattern. Interestingly, the metabolomic pattern of group A was closer to the control group than the other two groups (Figure 7).

**FIGURE 8:**
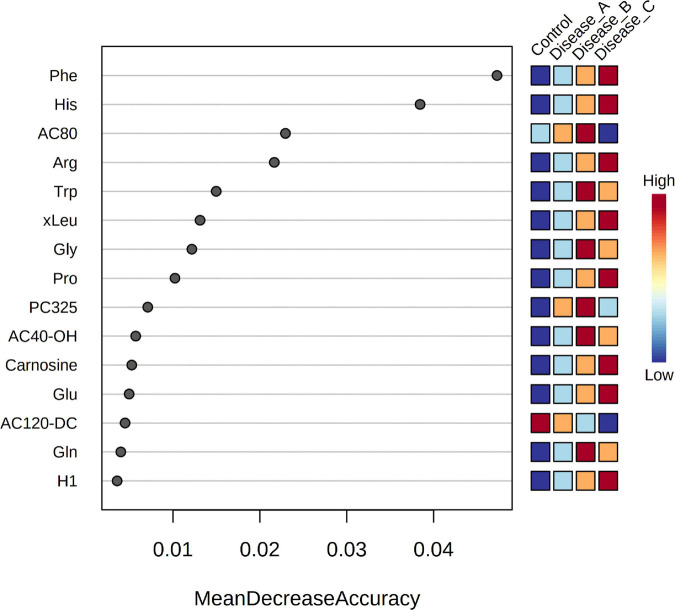
Random Forest variable importance plot for the metabolites obtained by the targeted metabolomics approach. A higher value indicates the importance of that metabolite in predicting groups.

Random forest analysis identified the metabolites that had the highest discriminatory power between the four groups as phenylalanine, histidine, and octanoylcarnitine (AC8:0), arginine, and tryptophan (Figure 8). RF classification showed an excellent prediction of the control group, while the classification of the affected groups showed less accuracy with a 35.3% OOB error rate. In targeted metabolomics, a high degree of overlap between the VIP scores and RF was found (11 out of 15 compounds).

Correlations between the metabolites identified by targeted approach and UPC (conventional parameter) are shown in the Supplementary Table 3. Five metabolites showed strong or very strong positive correlations with UPC: phenylalanine, proline, carnosine, arginine, and (iso)leucine (*r* ≥ 0.7, *p* < 0.05).

**FIGURE 9:**
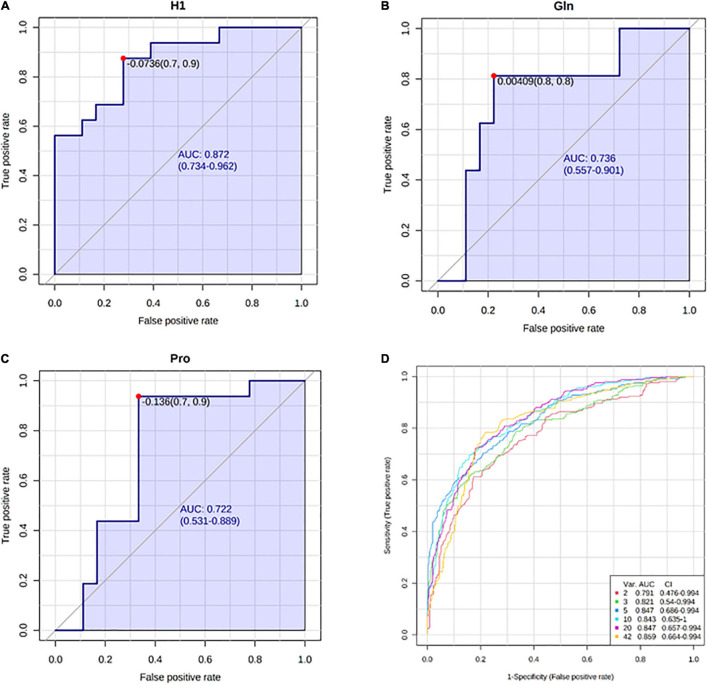
Receiver operating characteristic (ROC) curves with the corresponding area under curve (AUC) and 95% confidence intervals (CI) for: **(A)** hexoses (H1), **(B)** glutamine (Gln), **(C)** proline (Pro), **(D)** comparison of all models generated by multivariate ROC analysis.

Receiver operating characteristic curve analysis for individual features showed the best performance for hexoses (AUC = 0.872, *p* < 0.05), glutamine (AUC = 0.736, *p* < 0.05), and proline (AUC = 0.722, *p* < 0.05) in detecting kidney dysfunction (Figures 9A–C). Multivariate exploratory ROC analysis using the Support Vector Machine (SVM) as a classification method provided a comparison of all models showing that the model with only five features [H1, Arg, Gln, AC(14:1-DC), Pro] gives a relatively good performance (AUC = 0.847, 95% CI = 0.686–0.994; Figure 9D).

### Pathway Analysis and Enrichment Analysis

**FIGURE 10:**
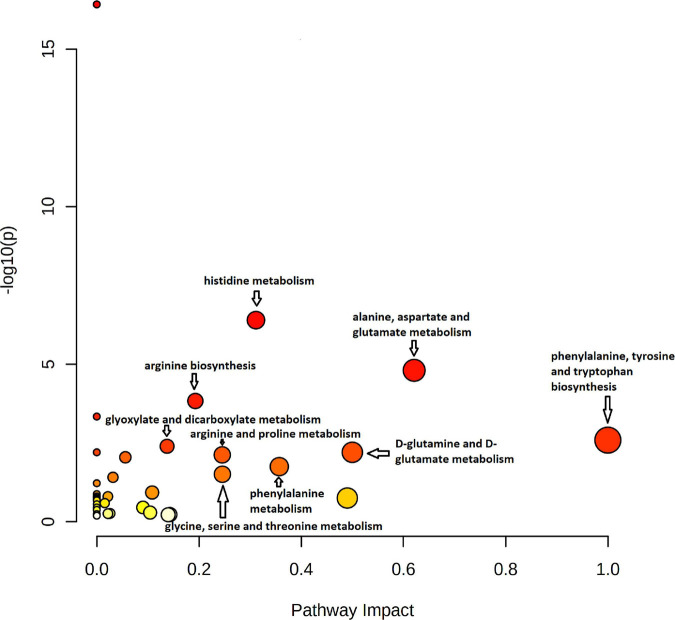
Pathway impact analysis plot of the significant metabolites identified by both untargeted and targeted approaches. The radius of the circle represents the number of metabolites identified in each pathway. The color gradient from white to red is representative of the cumulative statistical significance of the pathway and the individual pathway impact on the overall metabolic change observed. Pathways with *p* < 0.05 and impact >0.1 were considered to be significant.

Pathway analysis of 43 significant metabolites (H1 was excluded as this represents hexoses in general) identified by untargeted and targeted MS-based metabolomics approaches, showed nine different pathways considered to be significant with *p* < 0.05 and impact >0.1 (Figure 10 and Supplementary Table 4). The identified pathways were phenylalanine metabolism; glycine, serine, and threonine metabolism; histidine metabolism; alanine, aspartate, and glutamate metabolism; arginine biosynthesis; phenylalanine, tyrosine, and tryptophan biosynthesis; glyoxylate and dicarboxylate metabolism; D-Glutamine and D-glutamate metabolism; and arginine and proline metabolism.

**FIGURE 11:**
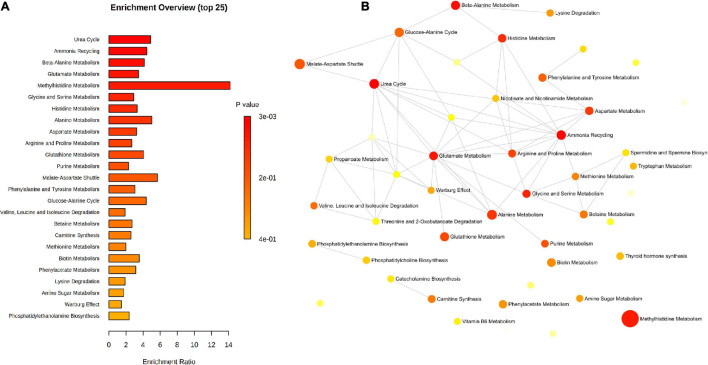
Pathway enrichment of the significant metabolites identified by untargeted and targeted approaches using the metabolic datasets. **(A)** Barchart view, **(B)** network view.

Pathway enrichment analysis using the metabolic sets showed that key metabolites participated in urea cycle, ammonia recycling, and amino acids metabolism (Figure 11).

## Discussion

In this study, we have shown that kidney dysfunction accompanying canine babesiosis was associated with alterations in the urinary metabolomics profile. Combining high resolution MS-based analytical platform for untargeted and targeted approaches, we obtained insight into the metabolomic signatures and associated pathways in canine babesiosis. Untargeted approach identified 601 features with a differential abundance between the healthy group and groups of dogs with babesiosis and different level of kidney function, with 27 of them identified as a match to known standards; while targeted approach identified 17 metabolites with significantly different concentrations between groups. Urine metabolites, directly affected by the functions of different tissues and organs, are excellent indicators of the metabolic status and can reveal the metabolic changes during disease (Liu et al., 2016). Moreover, kidney dysfunction has an additive effect in the urine composition at the metabolome level. Changes of the urinary metabolites excretion can reflect their plasma concentration, glomerular filtration rate, and intra-renal production and/or consumption (Zhang et al., 2015).

Amino acids (AA) metabolism is associated with the alterations of protein biosynthesis, occurrence of inflammation, and other conditions (Yu et al., 2020). The kidney is an important site of AA and protein metabolism, with an almost complete tubular AA reabsorption (Garibotto et al., 2010). Clinical conditions associated with kidney dysfunction, such as inflammation or metabolic acidosis, have been shown to affect AA and protein metabolism (Suliman et al., 2005; Duranton et al., 2014). Furthermore, with the progression of kidney damage, filtration, and reabsorption may be altered and lead to AA losses or proteinuria (Duranton et al., 2014).

Our study revealed alterations in the urinary amino acid profile, identifying significant changes in 13 AA, with six of them identified by both approaches. All essential AA (Phe, Thr, Trp, Met, Lys, His, Val, and xLeu) were significantly changed in our study, and these AA are considered as general markers for protein degradation as, assuming a steady dietary intake, variations in their concentrations are attributable to the catabolism of existing proteins due to tissue destruction, apoptosis, or autophagy (Fitzpatrick and Young, 2013). The aromatic amino acids (AAA), phenylalanine, tyrosine, and tryptophan, showed higher concentrations in dogs with babesiosis compared to healthy animals, with an excellent discrimination between the groups with different level of kidney function. These amino acids and their metabolism are linked to the synthesis of a diverse secondary metabolites, that have integral and key roles in animals (Parthasarathy et al., 2018). Chronic kidney failure is associated with significant alterations in the degradation, synthesis, or urinary excretion of the AAA, as demonstrated by human and animal studies (Kopple, 2007). Phenylalanine is involved in the protein synthesis and the formation of tyrosine under the action of hepatic and renal enzyme phenylalanine-4-hydroxylase (PAH). Previous study reported an increase of plasma phenylalanine concentration in inflammatory diseases (Wannemacher et al., 1976). Phenylalanine metabolism could be affected through the impairment of PAH activity due to immune activation and inflammation (Scholl-Bürgi et al., 2011). Increase of reactive oxygen species (ROS) during inflammatory conditions can cause irreversible oxidation of PAH cofactor, as well as influence on the tertiary structure of PAH resulting in an impairment of the substrate and/or the cofactor binding (Fuchs et al., 2014).

In babesiosis, pathophysiological mechanism of the disease could be mainly attributed to the host inflammatory response to the parasite. Acute phase response during babesiosis is well documented (Lobetti et al., 2000; Ulutas et al., 2005; Matijatko et al., 2007). Furthermore, production of pro-inflammatory cytokines and release of ROS have been linked to the endothelial cell activation and oxidative stress (Barić Rafaj et al., 2013; Zygner et al., 2014; Goddard et al., 2016; Crnogaj et al., 2017; Galán et al., 2018; Leisewitz et al., 2019). Tissue hypoxia, with its consequent nephrotoxic effect, is considered as a cause of kidney damage in dogs with babesiosis (Irwin and Hutchinson, 1991; Máthé et al., 2007). Increase in the serum concentration of proinflammatory cytokine tumor necrosis factor alpha (TNF-α) followed by the induction of vasodilatation leads to hypotension, ischemia, and kidney dysfunction (Zygner et al., 2014). C-reactive protein, an acute phase protein, was higher in all groups of dogs with babesiosis compared to controls, indicating an active inflammatory state in babesiosis. In the targeted approach, phenylalanine showed the highest discriminatory power between the investigated groups, as evidenced by the random forest analysis and VIP score. Similar pattern was found in the untargeted approach, confirming a consistency of both approaches. Alterations of the AAA profile in babesiosis could be associated with ongoing inflammation, oxidative stress, and tissue hypoxia causing kidney dysfunction. These findings are further highlighted by the related significant pathways identified in the study, such as phenylalanine, tyrosine, and tryptophan biosynthesis, and phenylalanine metabolism.

Alterations of arginine and proline metabolism, as well as arginine biosynthesis were also found significant between healthy dogs and dogs with babesiosis. In the study of the urine metabolomic profiles in patients with diabetic kidney disease, it was shown that metabolites related to arginine and proline metabolism have a correlation with renal function (Feng et al., 2020). Arginine is the precursor of proline, but also the only substrate for nitric oxide (NO) production and the precursor for other important immune molecules (Wu and Morris, 1998). Nitric oxide is the major mediator of the inflammatory host response and the marker of oxidative stress (Wu and Morris, 1998). Arginine is also necessary in activating the hepatic urea cycle for ammonia detoxification, which is proved by identifying urea cycle as the most significant metabolite set in this study. Therefore, urinary excretion of arginine could be related to the inflammatory response in babesiosis and consequent renal damage.

Increased proline excretion could be a response to an increased synthesis or decreased catabolism by the enzymes proline oxidase or proline dehydrogenase (PRODH), respectively. High lactate concentrations may lead to albuminuria by increasing proline concentration, as lactate is an inhibitor of PRODH (Elsherif et al., 2019). Increased lactate concentrations were reported in canine babesiosis caused by *B. rossi* (Nel et al., 2004) and *B. canis* (Torti et al., 2020), while an increased urinary albumin suggested glomerular damage in canine babesiosis (Kuleš et al., 2018). The described mechanism of proline metabolism associated with albuminuria could explain the increasing proline levels in the urine of dogs with babesiosis and different levels of kidney function.

Significant changes in the urinary metabolome were found in glutamine and glutamate metabolism between healthy dogs and dogs with babesiosis. Most of glutamine is broken down to glutamate and ammonium ion (NH_4_) and then used as a substrate for urea and glucose synthesis or for ATP production (Watford, 2015). Glutamate can be donated by various amino acids obtained from the catabolism of amino acids (Cruzat et al., 2018). Furthermore, glutamine plays an important role in cell proliferation, tissue repair process activity, and intracellular pathways associated with pathogen recognition, as it leads to the expression and production of inflammatory cytokines. Glutamine (via glutamate), cysteine, and glycine are the precursors for the synthesis of glutathione (GSH), indicating its role in oxidative stress (Mills et al., 2017).

In one study, changes in the glutamine levels were associated with the increased metabolic activity as a result of hypoxia found in the tumor environment (Eigenbrodt et al., 1998). Increased glutamate levels were found in the urine of patients with CKD, explained by a mechanism activated by metabolic acidosis due to the kidney function decrease (Posada-Ayala et al., 2014). Increased glutamate availability help excrete the acid load through the glutamine metabolism and urea cycle.

In previous studies, the majority of dogs with babesiosis were reported with metabolic acidosis and increased lactate concentrations (Leisewitz et al., 2001; Nel et al., 2004; Zygner et al., 2012). Canine babesiosis causes hemolytic anemia resulting in impaired oxygen delivery, generation of the lactate through anaerobic metabolism, and compromised buffering capacity of the blood. Excessive release of inflammatory mediators and cytokines are the major features of the pathophysiology in babesiosis, together with the present oxidative stress (Taboada and Merchant, 1991; Jacobson and Clark, 1994). These are all factors that can contribute to the observed changes in the glutamine and glutamate levels in the urine. Contribution of renal hypoxia could be demonstrated by the differences between groups A and B.

Branched-chain amino acids (BCAAs), valine, leucine, and isoleucine, had higher concentrations in the diseased groups compared to the healthy group, and, furthermore, these metabolites could also differentiate between the groups with different levels of kidney function. High concentrations of BCAAs are associated with oxidative stress and inflammation in different pathological conditions. One study found that BCAA can activate a mechanism that involves ROS generation and NF-κB activation in circulating peripheral blood mononuclear cells (Zhenyukh et al., 2017). Furthermore, elevated BCAA levels promote inflammation and oxidative stress in endothelial cells, enabling inflammatory cells adhesion and endothelial dysfunction (Zhenyukh et al., 2018). Activation of the NF-κB signaling pathway results in the release of pro-inflammatory molecules, such as interleukin-6, TNF-alpha, intracellular adhesion molecule-1 (ICAM-1), or IL-8, as previously reported in babesiosis (Barić Rafaj et al., 2013; Zygner et al., 2014, 2015; Goddard et al., 2016; Galán et al., 2018). Alterations of BCAAs, could, therefore, contribute to the inflammation and oxidative stress in babesiosis.

Another affected pathway in our study was the alanine, aspartate, and glutamate metabolism. Increased levels of alanine were observed in dogs with babesiosis with a gradual increase corresponding to the kidney dysfunction grade. This finding suggests that lactic acid was converted to alanine as a consequence of an increased glycolysis pathway activity during the course of babesiosis. This is consistent with recent observations in malaria patients, with which *Babesia* shares a phylogenetic proximity and various clinical manifestations of the disease (Abdelrazig et al., 2017). Moreover, alanine is also a precursor for gluconeogenesis in the liver, therefore, a higher level of alanine may also be an indicator of hepatic dysfunction in babesiosis.

Glycine is known as an inhibitory neurotransmitter in the central nervous system; however, different studies demonstrated that glycine has anti-inflammatory and cytoprotective effects through the inhibition of glycine-gated chloride channels and direct effects on target cells, such as the activation of macrophages and neutrophils inhibition (McCole, 2010). Increased concentrations of glycine were found in the urine of patients with inflammatory bowel disease (IBD) and diabetic nephropathy in the active phase (Dawiskiba et al., 2014; Shao et al., 2020).

In our study, we identified the different components of histidine metabolism in urine: higher levels of histidine and carnosine and lower levels of imidazole-4-acetate and *N*(pi)-methyl-L-histidine (3-methylhistidine) were found in dogs with babesiosis compared to the dogs in the control group. Histidine has functions in proton buffering, metal ion chelation, ROS scavenging, histaminergic system, and others (Holeček, 2020). Histidine is also a precursor of carnosine (β-alanyl-L-hystidine) synthesized by carnosine synthase from histidine and β-alanine. Carnosine acts as an antioxidant and it is related to the detoxification from free radicals and the by-products of membrane lipids peroxidation. This scavenger capacity was evident in different inflammatory diseases (Andrea et al., 2005). It was suggested as the biomarker of CKD in dogs, as a consequence of oxidative stress in the kidney, increased muscle catabolism, and impaired reabsorption due to tubular epithelium damage (Ferlizza et al., 2020). Previous study also suggested 3-methylhistidine as a metabolite able to distinguish CKD patients from healthy subjects (Zhang et al., 2015, 2016). Imidazole-4-acetate is a histidine and histamine metabolite, derived from the transamination of histidine or oxidation of histamine, a potent inflammatory mediator (Branco et al., 2018). Therefore, changes in the histidine metabolism components could be attributed to the inflammation and oxidative stress in babesiosis, with more announced changes in the advanced stages of kidney dysfunction demonstrating kidney injury in babesiosis.

Aspartate, arginine, alanine, glutamate, and glutamine are all associated with the urea cycle and ammonia detoxication activity, as demonstrated by pathway enrichment analysis. The catabolism of proteins results in the local and systemic accumulation of ammonia. As a toxic metabolite, ammonia is converted to urea, predominantly in the liver and kidney, in order to be excreted by urine (Morris, 2002). Alanine and glutamine are the major transporters of nitrogen in the blood, while arginine is synthesized as a product of the urea cycle. Nitrogen enters the urea cycle in the form of aspartate and NH4+, produced by transamination and deamination reactions from amino acids in the liver (Morris, 2002). Previous research demonstrated that the amino acids associated with the urea cycle were related to the inflammatory biomarkers and oxidative stress (Pietzner et al., 2017; Cao et al., 2019).

In CKD, total urinary AA increased with proteinuria, and there were significant correlations between proteinuria and urinary excretion of proline, asparagine, valine, alanine, threonine, lysine, phenylalanine, methionine, serine, tyrosine, and tryptophan (Duranton et al., 2014). The levels of valine, histidine, glycine, asparagine, and aspartic acid found in urine samples from patients with diabetic nephropathy were also increased, pointing that urine amino acid metabolism is associated with the development of nephropathy (Shao et al., 2020). Our study also confirms an increased urinary excretion of all these AA except serine. Additionally, levels of alanylglycine (Ala-Gly) were found higher in group C compared to all the other groups. Ala-Gly is a dipeptide found in the urine as a breakdown product from endogenous and exogenous proteins, therefore, its excretion could be a consequence of an increased protein catabolism and renal involvement in babesiosis.

Altered AA urinary effect in canine babesiosis is most likely related to the catabolic host response to infection, however, considering the role of the kidneys in AA and protein metabolism, kidney dysfunction progressively affects the urinary excretion of AAs. Considering the changes related to renal damage (groups B and C), according to ANOVA and *post hoc* test, as well as random forest analysis, correlations, ROC curves, and considering both approaches, phenylalanine, proline, histidine, glutamine, and arginine are of special interest due to their metabolomics pattern.

Another class of metabolites showing alterations in our study were acylcarnitines (AC). Acylcarnitines are acyl esters of carnitine, with functions in central carbon and lipid metabolism within the mitochondria. They are excreted through the urine as toxic metabolites and their accumulation is a consequence of mitochondrial beta-oxidation dysfunction (Reuter and Evans, 2012). Medium and short-chain fatty acid can enter the mitochondria directly, while long-chain fatty acids require enzyme assistance.

Acylcarnitine profile reflects changes in energy metabolism. The main source of energy for the proximal renal tubular cells is beta-oxidation of fatty acids (Feng et al., 2020). Compared to short-chain ACs that are produced from glucose, amino acids and fatty acid breakdown, medium- and long-chain ACs are derived only from fatty acid metabolism (Zhang et al., 2015). Therefore, concentrations of acylcarnitines with different chain lengths could differentiate the energy metabolism pattern (Makrecka-Kuka et al., 2017). Recently, it was shown that medium-chain acylcarnitines are able to activate the proinflammatory signaling pathways (Rutkowsky et al., 2014). Increased levels of free fatty acids in urine are shown to indicate renal tubular interstitial damage (Sasaki et al., 2009; Feng et al., 2020).

Altered fatty acid metabolism and incomplete beta-oxidation prevent the removal of excess lipids in the cells, resulting in increased levels of acylcarnitines in babesiosis. These metabolites are linked to the mitochondrial function and suggest an impairment of mitochondrial activity due to the kidney dysfunction. A similar mechanism of mitochondrial dysfunction was reported in different metabolic diseases (Bjørndal et al., 2018).

Urinary levels of two TCA cycle-associated metabolites, citrate and malonate, were significantly altered in our study. Citrate blood and urine levels are considered to reflect the TCA cycle activity considering a lack of extra-renal elimination and minimal influence by oral intake (Hamm, 1990). However, the acid-base status can affect the citrate renal excretion (Hallan et al., 2017).

In CKD, the urinary excretion of citric acid cycle metabolites and their renal gene expression were reduced (Hallan et al., 2017). Increased urine levels of TCA cycle intermediates, including citrate, were found in mice with diabetes, which might be the consequence of the systemic response caused by hyperglycemia or the local response on tubular transport and/or mitochondrial function in kidneys (Salek et al., 2007).

Malonate is a competitive inhibitor of succinate dehydrogenase (SDH), the only enzyme that participates in both the TCA cycle and the electron transport chain. These findings indicate a higher carbon flux through the TCA cycle accompanied by a reduced SDH activity. This increased TCA cycle flux may be related with the hypoglycemia previously demonstrated in canine babesiosis (Keller et al., 2004; Rees and Schoeman, 2008). Changes in the TCA cycle support a disruption in the energy metabolism in babesiosis.

Mitochondria are key organelles for energy production, including the complex metabolic processes such as the TCA cycle, oxidative phosphorylation, and beta-oxidation of fatty acids. In addition, mitochondria also play important roles in the regulation of inflammation (Mills et al., 2017). Several mitochondrial proteins, including the uncoupling protein 3 involved in oxidative phosphorylation, were also found in the urinary proteome of dogs with babesiosis and kidney injury (Winiarczyk et al., 2019). The ability to switch between available energy substrates is essential for cell homeostasis (Zhang et al., 2015). Our data indicated an alteration of mitochondrial activity in canine babesiosis and urine metabolomics profile in different groups of kidney function suggesting changes of the energy metabolism pattern due to renal damage.

Purines and pyrimidines are involved in immense biological processes, such as RNA and DNA synthesis, signal transduction and translation, energy metabolism modulation, and as structural components of various coenzymes (Jurecka, 2009; Maiuolo et al., 2016). During inflammation, metabolic imbalance, and malignant diseases, the turnover of RNA is faster than in normal body conditions, resulting in an altered nucleoside excretion in the urine (Schram, 1998). Receptors for adenine, an extracellular signaling molecule, are expressed in many organs, including the kidney (Santos et al., 2019). Renal adenosine levels increase in response to an excessive transport activity or impaired perfusion, leading to a GFR reduction (Vallon et al., 2006; Santos et al., 2019). Adenine and cytosine were detected in urine samples from mice in the LPS-induced inflammation model (Laiakis et al., 2012). Moreover, clinical studies have shown that in patients with CKD, the circulating levels of adenine is increased (Slominska et al., 2002).

Increased levels of adenine and its derivative hypoxanthine in urine could be attributed to the higher extent of RNA turnover due to infection and their local effect in the kidney. Increase of hypoxanthine was more profound in groups B and C, indicating a renal origin. On the other hand, cytosine levels were lower in dogs with babesiosis, regardless of the degree of kidney damage.

The kidney plays a role in the bile acid metabolism. Bile acids are the end products of cholesterol catabolism (Chiang, 2004). Conversion of cholesterol to bile acids is necessary for preserving cholesterol homeostasis and preventing a raise of lipids and toxic metabolites (Chiang, 2013). Bile acids are also metabolic regulators of lipid and glucose homeostasis in the enterohepatic system (Chiang, 2013). The bile acids filtered through the glomeruli are nearly completely reabsorbed due to an efficient tubular reabsorption process mediated mainly by the sodium-dependent bile acid transporter (ASBT), avoiding bile acid losses with the urine (Barnes et al., 1977; Wilson et al., 1981; Chiang, 2013). Previous studies revealed that downregulation of the ASBT in the proximal tubular cells leads to an increased urinary bile acid excretion (Schlattjan et al., 2003, 2005). It was shown that protein kinases, such as mitogen-activated protein (MAP) kinases, are involved in the regulation of the ASBT-mediated taurocholate uptake into proximal tubular cells, as well as on a sodium-independent tubular taurocholate transporter (Schlattjan et al., 2005). Within the repertoire of signaling molecules in the network of protein kinases, MAP kinases are also involved in conducting cellular responses to a diverse stimulants, as the host response to an infection (Pearson et al., 2001). The increase in taurocholate excretion in our study may result from an enhanced tubular secretion due to the changes of transporters activity during the course of babesiosis.

Vitamin B6 refers to six interconvertible compounds, vitamers, with vital roles in the cell metabolism. They are vital for enzymes linked to energy metabolism in the TCA cycle, gluconeogenesis, and in the one carbon metabolism pathway, for DNA synthesis, repair, and epigenetic regulation, and homocysteine metabolism (Bor et al., 2003). Pyridoxal is the transport form of vitamin B6, suggested as one of the markers of vitamin B6 status, and also highly correlated with pyridoxal-5’-phosphate (PLP), an active coenzyme form. Several studies have demonstrated the inverse relations of vitamin B6 with inflammatory diseases and oxidative stress, including surgery, trauma, inflammatory bowel disease, rheumatoid arthritis, diabetes, and cardiovascular diseases (Chiang et al., 2003, 2005; Huang et al., 2010; Keles et al., 2010; Kiran et al., 2011; Dhalla et al., 2013; Selhub et al., 2013). Besides inflammation, impaired kidney function is also reported to influence the B6 status (Ueland et al., 2015). PLP is required for transaminase-catalyzed reactions, such as transamination to glutamate and other amino acids, but also for one carbon metabolism of glycine and serine (Ueland et al., 2015). Therefore, reduced levels of pyridoxal in the urine could be a result of an increased protein catabolism as evidenced by increased levels of different amino acids in the urine of dogs with babesiosis and increased consumption of this vitamin needed for enzymes reactions, as evidenced by the increased activity of AST.

Choline is a vitamin-like essential nutrient and a methyl donor involved in different processes, such as cell structure and messaging, fat transport and metabolism, and DNA and neurotransmitter synthesis. In dogs, an increased tubular excretion of choline begins when the endogenous plasma choline level is doubled (Acara and Rennick, 1973). Choline is also associated with the modulation of inflammation through the “cholinergic anti-inflammatory pathway” demonstrating an anti-inflammatory activity in restraining excessive inflammation (Parrish et al., 2006; Rosas-Ballina and Tracey, 2009). In our study, higher levels of choline in all groups of dogs with babesiosis compared to the controls might be related to the inflammatory host response to the parasite.

Metabolite L-gulono-1,4-lactone (GUL) is an intermediate in the ascorbic acid synthesis, which belongs to the antioxidants scavenging free radicals (Kaźmierczak-Barańska et al., 2020). Lower urinary GUL levels in dogs with babesiosis could be a consequence of an increased consumption due to oxidative stress in babesiosis.

*N*-acetylneuraminate (Neu5Ac) is a conjugate base of a *N*-acetylneuraminic acid, also known as sialic acid, an essential component of glycoproteins and glycolipids. Being a constituent of cell membranes, vascular damage leads to Neu5Ac shedding into the circulation (Gökmen et al., 2000). Increased urinary *N*-acetylneuraminic acid excretion was reported in AKI, due to the release of Neu5Ac from sialoproteins in podocytes and tubular cells, and upregulated gene expression of a key synthesis enzyme (Martin-Lorenzo et al., 2017). Elevation of this metabolite in urine is related to kidney vascular cell damage and suggests renal tubular injury (Racine et al., 2004; Prajna et al., 2013). Inflammatory acute phase response and hemolytic anemia induced by babesiosis provoke endothelial cell activation and damage, vascular permeability increase, and tissue hypoxia, which may be associated to an increased shedding of Neu5Ac. Higher levels in group C suggest renal involvement and possible tubular injury.

Strengths of this study include the use of up-to-date complementary metabolomics approaches and methodological advantages of the urine sample as a simple non-invasive collection and richness in metabolites. The main study limitation is the small sample size, however, the large observed differences, consistency with similar findings, and consistency between the two approaches performed provided confidence in our results.

In conclusion, we observed a pattern of significantly altered metabolites referring to the inflammatory host response, oxidative stress, and energy metabolism modulation in babesiosis. Our findings have demonstrated that kidney dysfunction accompanying canine babesiosis was associated with changes in the amino acid metabolism, energy metabolism, fatty acid metabolism, and biochemical pathways linked to kidney function such as the urea cycle and ammonia detoxication. The results of this study provide a global overview of the urinary metabolome in response to *B. canis* infection in dogs and highlight new targets for further investigation. These findings will enable the inclusion of urinary markers for the detection and monitoring of renal damage in babesiosis, as well as in other similar diseases.

## Data Availability Statement

The original contributions presented in the study are included in the article/Supplementary Material, further inquiries can be directed to the corresponding author.

## Ethics Statement

The animal study was reviewed and approved by Committee on the Ethics of the University of Zagreb, Faculty of Veterinary Medicine (Permit Number: 640-01/18-17/63). Written informed consent for participation was not obtained from the owners because the samples used for the study were leftovers from usual clinical work.

## Author Contributions

VM, RB, and DE conceived and designed the experiments. PB, MB, and BB collected the samples. IR, JK, RB, and BB performed the experiment. JK and IR analyzed the data and wrote the manuscript. All authors have contributed to the article and approved the submitted version.

## Conflict of Interest

The authors declare that the research was conducted in the absence of any commercial or financial relationships that could be construed as a potential conflict of interest.

## Publisher’s Note

All claims expressed in this article are solely those of the authors and do not necessarily represent those of their affiliated organizations, or those of the publisher, the editors and the reviewers. Any product that may be evaluated in this article, or claim that may be made by its manufacturer, is not guaranteed or endorsed by the publisher.

## Supplementary Material

Supplementary Table 1 Untargeted metabolomics of urine in dogs with babesiosis and different level of kidney function. (A) Peak intensity table. (B) All features (identified and annotated) with differential abundance between groups identified by one-way analysis of variance (ANOVA) and Fisher’s LSD *post hoc* test. (C) Significant features identified based on retention times/masses or masses of detected peaks match to the standards.

Supplementary Table 2 Targeted metabolomics of urine in dogs with babesiosis and different level of kidney function. (A) Concentrations of all valid analytes obtained by AbsoluteIDQ^®^ p400 HR Kit (Biocrates Life Sciences AG, Innsbruck, Austria). (B) Metabolites with differential abundance between groups identified by ANOVA and Fisher’s LSD *post hoc* test.

Supplementary Table 3 Correlations between metabolites identified by targeted approach and urinary protein to creatinine ratio (UPC) showing: (A) Pearson’s correlation coefficients and (B) *p* values.

Supplementary Table 4 Pathway analysis of significant metabolites identified by untargeted and targeted MS-based metabolomics approaches.
